# Neuromuscular electrical stimulation for early rehabilitation in critically ill patients: a systematic review of applied protocols

**DOI:** 10.1186/s13054-026-05873-6

**Published:** 2026-02-10

**Authors:** Nils Daum, Nils Drewniok, Annika Bald, Laura Homann, Linus Warner, Flora T. Scheffenbichler, Antonia Leder, Max Liebl, Anett Reißhauer, Tobias Wollersheim, Stefan J. Schaller, Steffen Weber-Carstens, Julius J. Grunow

**Affiliations:** 1 Department of Anesthesiology and Intensive Care Medicine (CCM/CVK), Charité – Universitätsmedizin Berlin, Corporate Member of Freie Universität Berlin and Humboldt Universität zu Berlin, Berlin, Germany; 2 Institute of Medical Informatics, Charité – Universitätsmedizin Berlin, Corporate Member of Freie Universität Berlin and Humboldt Universität zu Berlin, Berlin, Germany; 3 Department of Anesthesiology and Intensive Care Medicine, Ulm University, Ulm, Germany; 4 Department of Anaesthesia, Intensive Care Medicine and Pain Medicine, Clinical Division of General Anaesthesia and Intensive Care Medicine, Medical University of Vienna, Vienna, Austria; 5 Division of Physical Medicine, Charité – Universitätsmedizin Berlin, Corporate Member of Freie Universität Berlin and Humboldt Universität zu Berlin, Berlin, Germany

**Keywords:** Neuromuscular electrical stimulation, Systematic review, Early rehabilitation, Intensive care, ICUAW

## Abstract

**Introduction:**

Neuromuscular electrical stimulation (NMES) is increasingly used to mitigate negative consequences of bed rest in critically ill patients, especially those unable to actively participate in mobilization. However, existing studies have shown inconsistent outcomes, potentially due to heterogeneous NMES protocols. This systematic review aimed to identify and characterize the NMES protocols applied in this context.

**Methods:**

A systematic literature search was conducted in MEDLINE, Cochrane Library, Pedro, and CINAHL up to August 18, 2025. Studies were selected based on predefined PICOS criteria, focusing on adult intensive care unit (ICU) patients receiving NMES for early rehabilitation. Only randomized controlled trials and observational studies were included. Data on protocol parameters and patient characteristics were extracted and descriptively analyzed.

**Results:**

44 studies with a total of 2,553 patients were included, applying 50 different NMES protocols. Stimulation predominantly targeted lower limb muscles using biphasic rectangular waveforms. Pulse duration ranged from 250–1,400 µs, frequencies from 20–121 Hz, and intensities from 2–250 mA or 20–250 V. Considerable heterogeneity was observed in stimulation settings, reporting methods, and protocol transparency.

**Conclusion:**

The reviewed studies highlight the lack of standardization in NMES protocols for ICU patients. Variability in stimulation parameters hinders comparability and clinical translation. Future research should aim to harmonize protocols respectively their reporting to strengthen the comparability of clinical studies and their outcomes.

**Graphical abstract:**

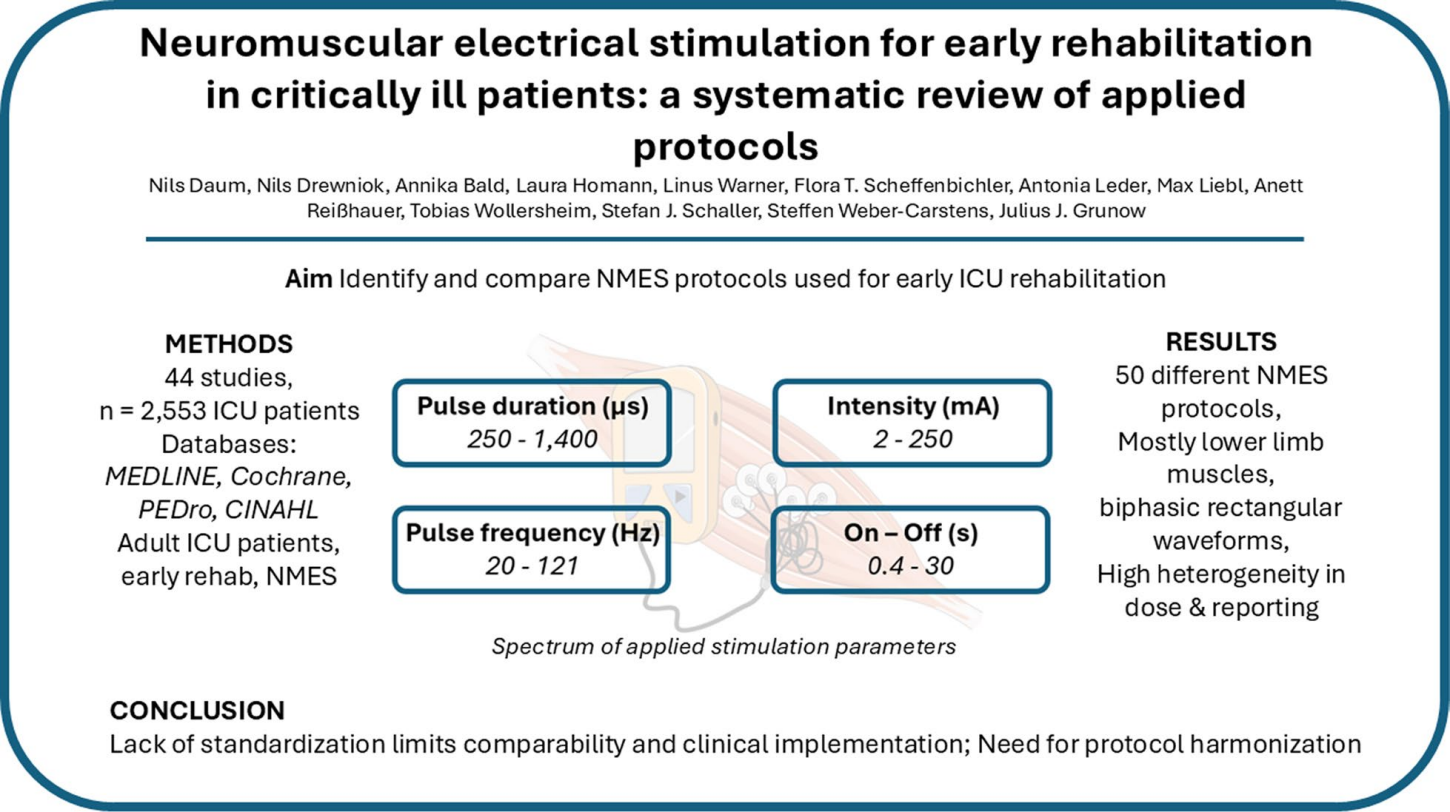

**Supplementary Information:**

The online version contains supplementary material available at 10.1186/s13054-026-05873-6.

## Introduction

Critical illness and intensive care unit (ICU) treatments are associated with short- and long-term physical impairments that substantially increase morbidity and mortality. Up to 80% of patients show physical impairments during the acute phase of critical illness [1], while increased mortality, delayed weaning from mechanical ventilation, prolonged hospitalization, and reduced quality of life are reported up to 5 years after discharge [2]. Therapeutic approaches to mitigate physical impairments include early mobilization. Early mobilization has been identified as a key intervention that can help prevent or mitigate the development of physical impairments by preserving neuromuscular function and promoting physical recovery in critically ill patients [3–7]. Additionally, early initiation of mobilization is crucial to positively impact the outcomes of critically ill individuals [8]. Active mobilization, as the most effective intervention, often encounters barriers in clinical practice, which are commonly linked to over-sedation [9, 10]. In these patients, active muscle contractions can be achieved through neuromuscular electrical stimulation (NMES), which employs electrical impulses to induce muscle contractions.

NMES has been incorporated into several international guideline recommendations as an adjunct to early mobilization [11–13]. Despite a considerable body of research, the available evidence remains inconsistent, which prevents the formulation of firm recommendations. This highlights a knowledge gap, as the effectiveness of NMES likely depends on standardized application. Studies and systematic reviews to date have yielded heterogeneous results, with some demonstrating clinical and molecular benefits while others showing no improvement [3, 8, 14–16]. Both EM and NMES share the commonality that the appropriate type and intensity of the intervention remain insufficiently studied [17, 18]. A potential source of the varying results might be the protocols used, which have never been systematically evaluated. Therefore, the aim of this systematic review was to identify and characterize different NMES protocols in ICU patients.

## Methods

### Protocol and registration

The protocol for this systematic review was registered on Open Science Framework (OSF) [19]. This study followed the Preferred Reporting Items for Systematic Reviews (PRISMA) reporting guidelines [20].

### Selection criteria

Before the start of the systematic search, inclusion criteria were established by common consensus using the PICOS (participants, interventions, comparisons, outcomes, and study design) criteria (Table 1). Only full-text, peer-reviewed articles were included. Grey literature, conference abstracts, and unpublished studies were not considered, as detailed protocol descriptions were required for the objectives of this review.

**Table 1:** PICOS criteria for the inclusion criteria of the systematic literature review

P	Critically ill patients (≥ 18 years old) who were admitted to the ICU
I	Neuromuscular Electrical Stimulation to improve muscle function or muscle mass
C	Standard care or no stimulation
O	Protocol Parameters (e.g. Duration, Number of Sessions, Intensity (Minimal acceptable response, Maximal limit), Frequency, On-/Off-Time, Wave Length, Wave Form, Monopolar/bipolar, Muscle groups stimulated)
S	Randomized controlled trials (RCT) or observational studies

Studies were excluded if they met either of the following exclusion criteria:

1. Studies that were not in the German or English language
2. NMES with the goal of improving range of motion, spasticity or cardiovascular function

### Information sources

The systematic search strategy was developed jointly by all authors, and after approval, the following databases were searched: MEDLINE via PubMed, Cochrane Library, Pedro, and CINAHL. The exact search strategy of the databases used is shown in Table A1 in the Appendix. The search extended from the inception of the respective database to August 18th, 2025. We additionally searched the reference list of review articles for relevant studies and added them manually.

### Selection of studies

Two reviewers independently screened all studies from the systematic search by title and abstract for PICOS criteria using Rayyan [21]. Full-text articles were subsequently assessed for eligibility. Each study received two independent and blinded assessments, and disagreements were resolved by a third independent reviewer.

### Data retrieval process

The included full texts were analyzed by one reviewer, and all relevant information was extracted. A second reviewer verified the accuracy of all transferred data. Data were obtained from both protocol descriptions and reported results whenever applicable, and any discrepancies between sources were resolved by consensus.

### Data details and risk of bias

All relevant PICOS criteria were extracted from the included studies. In addition, we extracted study- and patient-specific characteristics. A descriptive analysis of the studies was performed. The risk of bias was assessed using the RoB-2 tool [22] for randomized controlled studies (RCTs) and ROBINS-E tool [23] for prospective studies. Two raters independently evaluated each study. In case of discrepancies, a third rater determined the final overall risk of bias.

## Results

### Selection of studies

**Fig. 1:**
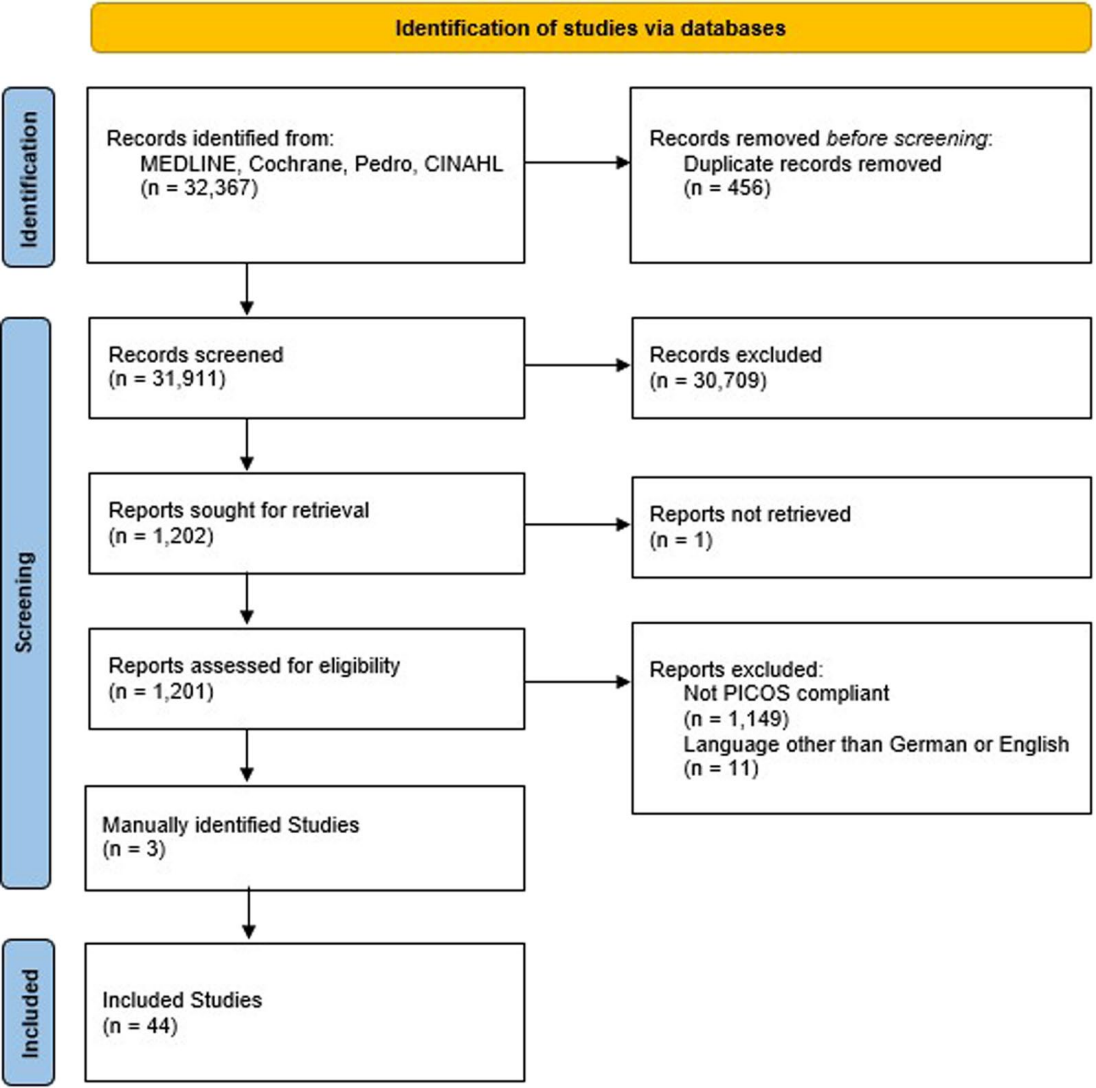
Flowchart of the systematic search

Of the 32,367 studies screened, 44 met the inclusion criteria and were included in the descriptive analysis (Fig. 1).

### Characteristics of included studies

A total of 40 included studies were designed as RCTs, including three that were explicitly conducted as pilot studies, and four as observational studies. Across these trials, a total of 50 distinct NMES protocols were applied to 2,553 patients. In five studies, two or more different NMES protocols were used concurrently (Table 2).

**Table 2:** Study characteristics

Author	Year	Study Design	Sample Size (n)	Study Arms	Intervention 1	Intervention 2	Intervention 3	Control
Abu-Khaber [24]	2013	RCT	80	interindividual	NMES			Usual care
Akar [25]	2017	RCT	30	interindividual	NMES + Physiotherapy	NMES only		Physiotherapy
Bao [26]	2022	RCT	60	interindividual	NMES (two muscles) + Physiotherapy	NMES (one muscle) + Physiotherapy		Physiotherapy
Baron [27]	2022	RCT	149	interindividual	NMES			Usual care
Berney [28]	2021	RCT	162	interindividual	NMES + Cycling			Usual care
Campos [29]	2022	RCT	74	interindividual	NMES			Usual care
Cerqueira [30]	2018	RCT	59	interindividual	NMES			Usual care
Dall’Acqua [31]	2017	RCT	25	interindividual	NMES			Sham NMES + usual care
Dirks [32]	2015	RCT	6	intraindividual	NMES			Usual care
Dos Santos [33]	2020	RCT	51	interindividual	NMES + Physiotherapy	Physiotherapy	NMES	Usual care
Falavigna [34]	2013	RCT	11	intraindividual	NMES			Usual care
Figueiredo [35]	2023	OBS	49	interindividual	NMES + Cycling			NMES + Cycling
Figueiredo [36]	2024	OBS	20	intraindividual	NMES + Cycling			None
Fischer [37]	2016	RCT	54	interindividual	NMES			Sham NMES
Fossat [38]	2018	RCT	312	interindividual	NMES + Cycling			Usual care
Gerovasili [39]	2009	RCT	49	interindividual	NMES			Usual care
Gruther [40]	2010	RCT	17	interindividual	NMES			Sham NMES
Guerra-Vega [41]	2025	RCT	52	interindividual	NMES (low frequency)	NMES (moderate frequency)		Usual care
Hirose [42]	2013	RCT	15	interindividual	NMES			Usual care
Karatzanos [43]	2012	RCT	52	interindividual	NMES			Usual care
Kho [44]	2015	RCT	34	interindividual	NMES			Sham NMES
Kourek [45]	2024	OBS	16	intraindividual	NMES			None
Koutsioumpa [46]	2018	RCT	80	interindividual	NMES			Usual care
Leite [47]	2018	Pilot RCT	50	interindividual	NMES			Usual care
Liu [48]	2023	RCT	80	interindividual	NMES			Usual care
Mahran [49]	2023	RCT	118	interindividual	NMES			Sham NMES
Medrinal [50]	2017	RCT	19	intraindividual	NMES + Cycling	NMES	Cycling	Usual care
Meesen [51]	2010	Pilot RCT	19	interindividual	NMES			Usual care
Nakamura [52]	2019	RCT	37	interindividual	NMES			Usual care
Nakanishi [53]	2020	RCT	36	interindividual	NMES			Usual care
Othman [54]	2023	RCT	120	interindividual	NMES + Physiotherapy	NMES alone	Physiotherapy	Usual care
Poulsen [55]	2011	RCT	8	intraindividual	NMES			Usual care
Rodriguez [56]	2012	RCT	14	intraindividual	NMES			Usual care
Routsi [57]	2010	RCT	140	interindividual	NMES			Usual care
Segers [58]	2021	RCT	47	intraindividual	NMES			Usual care
Silva [59]	2017	OBS	11	intraindividual	NMES			None
Silva [60]	2019	RCT	60	interindividual	NMES			Physiotherapy
Stefanou [61]	2016	RCT	32	intraindividual	NMES			NMES
Verceles [62]	2023	RCT	39	interindividual	NMES + High Protein Nutrition			Usual care
Vieira [63]	2023	RCT	40	interindividual	NMES			Usual care
Waldauf [64]	2021	RCT	150	interindividual	NMES + Cycling			Usual care
Wollersheim [65]	2019	RCT	50	interindividual	NMES ± whole body vibration			Usual care
Woo [66]	2018	Pilot RCT	10	intraindividual	NMES + Cycling			Cycling
Zulbaran-Rojas [67]	2022	RCT	16	interindividual	NMES			Usual care

*NMES *Neuromuscular Electrical Stimulation; *OBS* Observational study; *RCT* randomized controlled trial

Randomization was performed at the interindividual level in 33 studies, while ten studies implemented intraindividual randomization, for example, by applying different interventions to the right and left leg. In terms of study design, 37 studies employed a two-arm structure, three studies had a three-arm design, and three studies featured a four-arm design.

The most frequently investigated comparison was NMES in combination with standard care ("usual care") versus standard care alone. Three studies additionally compared both NMES plus standard care and NMES alone against standard care. In four studies, NMES was combined with passive or active cycling interventions; three studies specifically examined the additive effect of a combined NMES-cycling therapy. Further multimodal approaches included combinations of NMES with high-protein nutritional support, low- and moderate-frequency NMES, and whole-body vibration.

To control for potential placebo effects, five studies employed a sham NMES condition as a control. A detailed overview of the patient characteristics across studies is provided in Table A2 and A3 in the appendix.

### Characteristics of NMES protocols

**Fig. 2:**
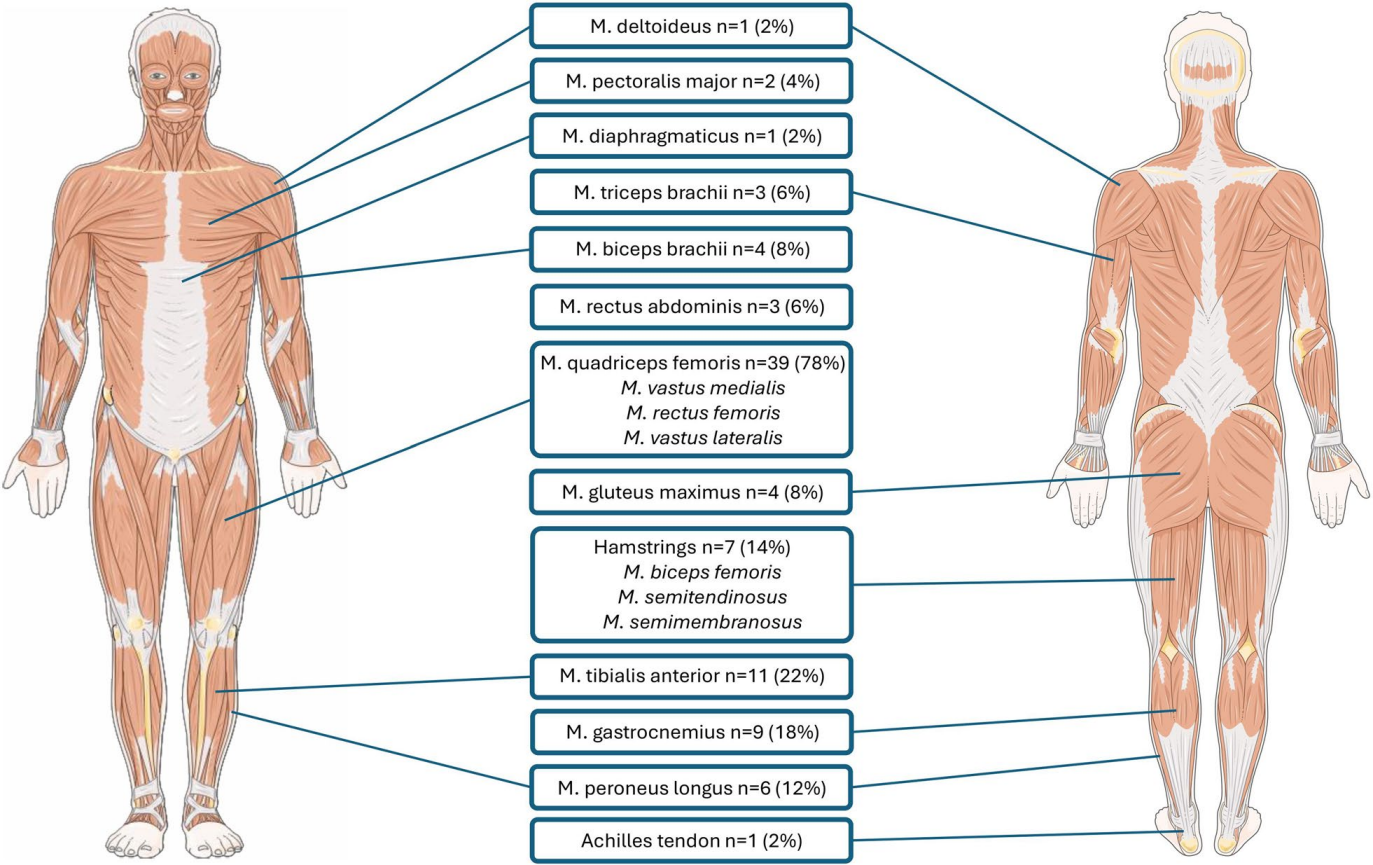
Absolute and relative frequencies of stimulation protocols stratified by the respective muscle groups targeted

Analysis of the NMES protocols revealed a predominant focus on stimulation of the lower extremity. The *quadriceps femoris muscle*, including its components *vastus lateralis*, *vastus medialis*, and *rectus femoris*, was most frequently targeted (n = 39 (78%)). Other regularly stimulated muscles included the *tibialis anterior* (n = 11 (22%)), *gastrocnemius* (n = 9 (18%)), *peroneus longus* (n = 6 (12%)), and *gluteus maximus* (4 (8%)) (Table 3 and Fig. 2). In select cases, stimulation also targeted trunk muscles, such as the *rectus abdominis* (n = 3 (6%)), or *upper limb muscles* (n = 9 (18%)), most commonly the *biceps brachii, triceps brachii*, and *pectoralis major* (Fig. 2.).

**Table 3.** NMES protocol parameters

Author	Year	Stimulated muscle	Waveform	Pulse duration (µs)	Pulse frequency (Hz)	min. intensity (mA)	max. intensity (mA)	mean intensity (mean)	On (s)	Off (s)	Minutes per day	Start after admission (days)	Number of stimulations / day	Number of sessions / patient
Abu-Khaber [24]	2013	Quadriceps	biphasic, symmetric	**200**	**50**	**100**	**150**	na	**15**	na	**60**	na	1	na
Akar [25]	2017	Deltoid / Quadriceps	biphasic, symmetrical, square	na	**50**	**20**	**25**	na	**6**	na	na	na	5	20
Bao [26]	2022	Gastrocnemius / Tibialis anterior	biphasic, asymmetrical, balanced rectangular	**300**	**30**	na	na	na	na	na	**40**	**2–3**	11–12	na
Baron [27]	2022	Gluteus maximus	symmetric biphasic rectangular	**500**	**100**	na	na	*45.8 (8.4)*	**5**	**25**	**25**	**2**	6	3 (2–5)
Berney [28]	2021	Rectus femoris / Hamstrings / Gluteus maximus / Gastrocnemius	na	na	na	na	na	na	na	na	**60**	na	na	5 (3–9)
Campos [29]	2022	Quadriceps / Tibialis anterior	Biphasic and symmetrical rectangular	**400**	**80**	na	**120**	na	**5**	**10**	**60**	** < 2**	5	na
Cerqueira [30]	2018	Gastrocnemius / Quadriceps	na	**400**	**50**	**17.5**^**a**^** / 22.8**^**b**^	**79.1**^**a**^** / 71.2**^**b**^	**49.5**^**a**^** / 54.9**^**b**^	**3**	**9**	**60**	**0**	5	10 (5–10)
Dall’Acqua [31]	2017	Pectoralis Major	na	**300**	**50**	na	na	*53 (15)*	**3**	**10**	**30**	na	7	na
Dall’Acqua [31]	2017	Rectus Abdominis	na	**300**	**50**	na	na	*68 (18)*	**3**	**10**	**30**	na	7	na
Dirks [32]	2015	Quadriceps / Rectus femoris / Vastus lateralis	biphasic, symmetric, rectangular wave pulse	**400**	**100**	na	na	*29.9–32.3*	**3.5**	**10**	**60**	**2.5**	3–10	na
Dos Santos [33]	2020	Rectus femoris / Vastus lateralis / Vastus medialis	biphasic symmetric	**400**	**45**	na	na	na	**12**	**6**	**110**	**2**	7.1	11.7
Dos Santos [33]	2020	Quadriceps	biphasic symmetric	**400**	**45**	na	na	na	**12**	**6**	**110**	**2**	5.8 (3.1)	10.3 (4.8)
Falavigna [34]	2013	Quadriceps / Tibialis anterior	biphasic, symmetric	**400**	**50**	*22*^*c*^* / 18*^*d*^	*60*^*c*^* / 48*^*d*^	*39.4*^*c*^* / 30.4*^*d*^	**9**	**9**	**20**	na	10.2 (9)	na
Figueiredo [35]	2023	Quadriceps / Tibialis anterior / Hamstrings	biphasic, rectangular	**500–1,000**	**50–100**	**50**	**250**	na	na	na	na	na	na	na
Figueiredo [36]	2024	Quadriceps / Tibialis anterior / Hamstrings	biphasic, rectangular	**500–1,000**	**50–100**	**50**	**250**	na	na	na	*11.3*	na	na	na
Fischer [37]	2016	Quadriceps	biphasic, rectangular	**400**	**66**	*2*	*100*	*40.5* *(2–100)*	**3.5**	**4.5**	**60**	*1*	4 (2–13)	na
Fossat [38]	2018	Quadriceps	na	na	**30–75**	na	na	na	**4–5**	**10–14**	**54**	na	na	na
Gerovasili [39]	2009	Quadriceps / Peroneus longus	biphasic, symmetric	**400**	**45**	**19**	**55**	**38 (10)**	**12**	**6**	**55**	**2**	na	na
Gruther [40]	2010	Quadriceps	biphasic, symmetric	**350**	**50**	na	na	na	**8**	**24**	**30**^** g**^** / 60**^** h**^	** < 7**	na	na
Guerra-Vega [41]	2025	Quadriceps	biphasic	**400**	**100**	*18.6*	*77.5*	*47.4*	**5**	**10**	**40**	**1**	2	na
Hirose [42]	2013	Quadriceps / Tibialis anterior / Biceps femoris / Triceps surae	na	na	na	**30**	**40**	na	**10**	**10**	**30**	**7**	7–14	na
Karatzanos [43]	2012	Vastus lateralis / Vastus medialis medialis, and peroneus longus	na	**400**	**45**	na	na	na	**12**	**6**	**55**	na	na	8 (6)
Kho [44]	2015	Quadriceps	biphasic, asymmetrical, balanced rectangular waveform,	**400**	**50**	na	na	na	**5**	**10**	**53**	*4.6 (1.8)*	na	na
Kho [44]	2015	Tibialis anterior / Gastrocnemius	biphasic, asymmetrical, balanced rectangular waveform,	**250**	**50**	na	na	na	**5**	**5**	**53**	*4.6 (1.8)*	na	na
Kourek [45]	2024	Vastus lateralis / Vastus medialis / Peroneus longus / Gastrocnemius	biphasic, symmetric	**400**	**75**	*30*	*86*	*53*	**5**	**21**	**45**	na	na	na
Koutsioumpa [46]	2018	Quadriceps	na	**500**	**50**	na	na	na	na	na	**60**	na	na	na
Leite [47]	2018	Vastus lateralis / Vastus medialis / Rectus femoris	na	na	**50**	na	na	na	**8**	**30**	**45**	na	na	na
Liu [48]	2023	Pectoralis major / Rectus abdominis / Quadriceps	na	**300**	**50**	na	na	na	**3**	**10**	**30**	na	1	na
Mahran [49]	2023	Rectus abdominis / Diaphragm	na	na	**30**	na	na	na	**1**	**20**	**20**	**2**	5	5
Medrinal [50]	2017	Quadriceps	biphasic, rectangular	**300**	**35**	na	na	na	na	na	**10**	na	na	na
Meesen [51]	2010	Rectus femoris / Vastus medialis	biphasic symmetric rectangular	**250–330**	**60–100**	na	**120**	na	**7–10**	**14–20**	**30**	**1**	1	na
Nakamura [52]	2019	All lower extremities	na	**250**	**20**	na	na	na	**5**	**2**	**20**	**2**	1	na
Nakanishi [53]	2020	Biceps brachii / Rectus femoris	square (positive) exponential decay (negative)	**650**	**20**	na	na	**30**^**e**^** / 41**^**f**^	**0.4**	**0.6**	**30**	na	5	na
Othman [54]	2023	Vastus medialis / Vastus lateralis / Rectus femoris	biphasic, asymmetrical, balanced rectangular	**400**	**50**	na	na	na	**5**	**10**	**60**	**2**	1	5
Poulsen [55]	2011	Quadriceps / Vastus medialis	biphasic	**300**	**35**	*23*	*48*	*31*	**4**	**6**	**60**	na	7	7
Poulsen [55]	2011	Quadriceps / Vastus lateralis	biphasic	**300**	**35**	*37*	*54*	*42*	**4**	**6**	**60**	na	7	7
Poulsen [55]	2011	Vastus medialis / Vastus lateralis	biphasic	**300**	**35**	na	na	na	**4**	**6**	**60**	**3 (1–4)**	na	na
Rodriguez [56]	2012	Quadriceps / Vastus medialis / Biceps brachii	biphasic	**300**	**100**	***20 V***	***200 V***	na	**2**	**4**	**60**	**2**	13 (7–30)	na
Routsi [57]	2010	Vastus lateralis / Vastus medialis / Peroneus longus	biphasic	**400**	**45**	na	na	na	**12**	**6**	**55**	**24–48**	1	na
Segers [58]	2021	Vastus medialis / Rectus femoris / Vastus lateralis	na	**350**	**45**	na	**120**	*75*	**8.5**	**12**	**60**	**2–4**	7	na
Silva [59]	2017	Gluteus maximus / Gastrocnemius / Hamstrings / Tibialis anterior / Quadriceps	bipolar rectangular	**1,000**	**100**	na	**69**	*52–57*	**5**	**5**	**15**	na	3	na
Silva [60]	2019	Quadriceps / Hamstrings / Tibialis anterior / Gastrocnemius	na	**400**	**100**	na	na	*65 (62–67)*	**5**	**25**	**25**	na	1	na
Stefanou [61]	2016	Vastus lateralis / Vastus medialis / Peroneus longus	biphasic, symmetric, trapezoid	**400**	**75**	na	na	na	**6**	**21**	**40**	na	7.6 (0.8)	1
Stefanou [61]	2016	Vastus lateralis / Vastus medialis / Peroneus longus	biphasic, symmetric, trapezoid	**400**	**45**	na	na	na	**5**	**12**	**40**	na	7.6 (0.8)	1
Verceles [62]	2023	Quadriceps / Dorsiflexor muscles of lower extremities	biphasic	**300**	**30**	na	na	na	**10**	**10**	**60**	** > 24**	0.8 (0.3)	10.1 (4.5)
Vieira [63]	2023	Rectus femoris	biphasic, symmetric, rectangular	**400**	**50**	na	na	na	**6**	**12**	**55**	** < 24**	5	5
Waldauf [64]	2021	Quadriceps	na	**250**	**40**	**0**	**60**	na	na	na	*31.1 (10.1)*	*2–3*	6.5	6.5
Wollersheim [65]	2019	Tibialis anterior / Triceps surae / Vastus lateralis / Biceps brachii / Triceps brachii / Wrist extensors / Wrist flexors	na	**350**	**50**	**40**	**70**	na	**6 or 10**	**10 or 15**	**20**	** < 3**	na	na
Woo [66]	2018	Quadriceps / Rectus femoris	biphasic symmetric square	**250**	**35**	na	na	na	**10**	**12**	**20**	na	na	na
Zulbaran-Rojas [67]	2022	Gastrocnemius / Achilles tendon	asymmetrical damped sinusoidal biphasic	**400–1,400**	**20–121**	***50 V***	***250 V***	na	na	na	**60**	**1.8 (1.9)**	1	14

^a^Gastrocnemius; ^b^Quadriceps; ^c^Protocol begin; ^d^Protocol end; ^e^Biceps brachii; ^f^Rectus femoris; ^g^First week; ^h^After first week; **Bold** Data extracted directly from the methodology *Italic* Data extracted from the results ***Bold italic*** Definition of the unit used deviates from the standard measure; *na* not available

Most studies (n = 26 (52%)) employed biphasic electrical stimulation, with symmetric rectangular or trapezoidal waveforms (n = 6 (12%)) being the most common. Three studies specifically described a biphasic-asymmetric, balanced rectangular waveform (n = 3 (6%)); trapezoidal or exponentially decaying waveforms were reported in two studies (n = 2 (4%)).

**Fig. 3:**
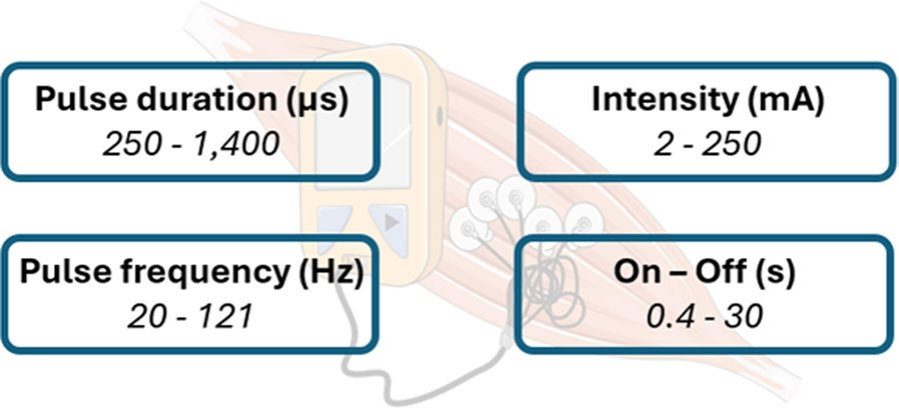
Comprehensive overview of the spectrum of stimulation parameter settings applied

Of the 50 NMES protocols identified, 44 (88%) explicitly reported the pulse duration in microseconds (µs), ranging from 250 to 1,400 µs. The stimulation frequency (Hz) was specified in 48 (96%) protocols, with values ranging from 20 to 121 Hz (Fig. 3).

Stimulation intensity (mA) was documented in 26 (52%) protocols, with reported values between 2 and 250 mA. Two studies reported intensity in volts (V), further complicating cross-study comparability.

The duration of stimulation and rest phases (on-/off-times) varied considerably: on-times ranged from 0.4 to 12 s, while off-times ranged from 0.6 to 30 s. Daily stimulation duration was reported in 47 (94%) protocols and ranged from 10 to 110 min per day. The number of stimulation sessions per day and per patient ranged from one to fourteen sessions.

### Risk of bias

The majority of included studies demonstrated a moderate to high risk of bias, with only three RCTs classified as having a low risk of bias (Table A4 and A5 in Appendix).

## Discussion

The present systematic review highlights a considerable heterogeneity in the NMES protocols employed across the included studies. Particularly striking is the wide range of stimulation parameters used including pulse duration (µs), frequency (Hz), intensity (mA), and on/off times, which substantially limits the comparability of results and hinders robust conclusions regarding the efficacy of specific protocols.

Several RCTs investigated the application of NMES in critically ill patients and reported beneficial effects on the preservation of muscle mass [16]. In many cases, the intervention groups demonstrated reduced muscular atrophy compared to their respective control groups. Some studies also observed a significant reduction in hospital length of stay. However, a consistent, evidence-based benefit regarding muscle strength or functional outcomes (e.g., mobility, rehabilitation duration) has not yet been clearly demonstrated [8, 15].

While various meta-analyses indicate potential positive effects of NMES on clinical endpoints, other studies found no significant advantage compared to EM strategies without NMES, especially regarding ICU length of stay [8, 14, 15]. A major reason for these inconsistencies could be due to the pronounced methodological heterogeneity among the primary studies. Not only did the NMES interventions differ—ranging from isolated application to combination with other measures such as in-bed cycling or physiotherapy—but the type of control group also varied widely (e.g., standard care vs. sham NMES).

Substantial differences have been reported in the technical implementation of NMES. Stimulation intensity, for example, has not only varied considerably in magnitude but has also been expressed in different units (mA vs. V), which complicates comparisons across studies. In addition, several investigations provided insufficient methodological detail, or descriptions had to be inferred from the results, limiting interpretability and reproducibility. This heterogeneity likely represents a major reason for the divergent findings observed in NMES trials, as variations in stimulation protocols appear to directly influence surrogate outcomes such as muscle strength or endurance [29, 41, 68]. To address this issue, a standardized NMES reporting sheet has been proposed to ensure transparent documentation of stimulation parameters and responder rates, thereby enabling systematic comparisons across patient cohorts and trials. Importantly, the induction of visible muscle contractions has been identified as a prerequisite for clinical efficacy. In one analysis, a stimulation intensity of 50.1 mA was sufficient to reliably elicit a contraction in responders, with a sensitivity of 100% and a specificity of 84.6% [68]. However, the magnitude of the evoked muscle force represents the primary determinant of NMES-induced strength adaptations, with stronger contractions being associated with greater training effects [69]. Accordingly, a merely visible contraction likely reflects a lower contraction quality. Based on the classification proposed by Segers et al., visible contractions correspond to a *Type 3* response, whereas higher-quality *Type 5* contractions, defined as strong, palpable, and clearly visible activation of the full muscle bulk, may represent a more appropriate stimulation target [60, 70]. Thus, relying solely on visible contractions as a dosing criterion may result in suboptimal stimulation intensity and contribute to heterogeneous clinical effects across NMES studies. Nevertheless, many published studies applied substantially lower intensities, raising concerns about whether effective muscle activation was consistently achieved. Taken together, these findings underscore the need for harmonized reporting standards and protocol consistency. Only by ensuring adequate stimulation thresholds and transparent documentation will it be possible to generate comparable data, and ultimately clarify the true clinical value of NMES in critically ill patients.

These findings are reflected in current clinical guidelines, which, due to a lack of robust evidence, do not provide specific parameters for recommendation [11, 12]. The stimulation approaches employed in the included studies were so heterogeneous that evidence-based standardization is currently not feasible. This heterogeneity also substantially limits the interpretability of pooled effect estimates in meta-analyses, as meaningful comparison or synthesis across protocols becomes inherently challenging.

In sport science and studies in healthy populations, NMES is applied with more consistent and purpose-specific protocols. For muscle hypertrophy and strength, high-frequency (50–100 Hz), high-intensity stimulation at maximally tolerable levels, and pulse durations between 200 and 1,000 μs are typically used, with sessions performed 3 times per week over several weeks [71, 72]. Endurance-focused protocols typically apply lower stimulation frequencies (10–30 Hz) and intensities combined with longer duty cycles, which are intended to promote fatigue resistance rather than selective fiber-type recruitment. Importantly, NMES-induced muscle activation does not follow the physiological size principle; instead, muscle fibers are recruited in a non-selective and asynchronous manner once depolarization occurs, with simultaneous activation of type I and type II fibers. Thus, differences between endurance- and strength-oriented NMES protocols likely reflect distinct metabolic and fatigue-related adaptations rather than preferential recruitment of specific muscle fiber types [73, 74].

Based on our findings and the literature in healthy individuals, it seems meaningful to explore NMES protocols using longer pulse durations and moderate- to high-frequency stimulation, with intensities individually titrated to elicit effective muscle contractions in ICU patients [75, 76]. Importantly, the term “wide-pulse” refers to pulse duration (µs) and should be interpreted in relation to individual neuromuscular excitability rather than as a fixed threshold, as critically ill patients, particularly those with ICU-acquired polyneuromyopathy, may require substantially prolonged pulse durations to achieve muscle depolarization [59, 77, 78]. As highlighted in Fig. 3, the parameter ranges reported in ICU studies span from very low to very high values (pulse duration 250–1,400 μs; frequency 20–121 Hz; intensity 2–250 mA; duty cycles 0.4–30 s), underscoring the urgent need for systematic testing of parameter combinations tailored to this population. Translational studies are therefore required to define safe and effective stimulation settings for critically ill patients, which may help bridge the gap between experimental protocols in sport science and the heterogeneous approaches currently applied in the ICU [16, 68, 70, 79]. Importantly, the choice of outcome measures must be aligned with the underlying stimulation strategy. For example, lower-frequency protocols (e.g., 20–30 Hz), which have demonstrated positive effects in ICU populations, may be more appropriately evaluated using functional or endurance-related outcomes, whereas higher-frequency stimulation is better assessed using objective, strength-related measures that quantify muscle force or contractile capacity, potentially also depending on the underlying pathophysiology (e.g., CIP vs. CIM) [60, 80].

Despite different application methods, NMES is generally considered a safe intervention in critically ill patients. Several studies that specifically evaluated feasibility and safety have reported a low incidence of adverse events and good tolerability of NMES in the ICU setting. Compared to other mobilization techniques, such as conventional physiotherapy or in-bed cycling, NMES appears to be associated with a favorable safety profile [38, 59, 81]. In view of its positive effects and favorable safety profile, NMES appears to be a reasonable option for supporting the early rehabilitation of critically ill patients [11, 12]. This is particularly relevant for patients who are unable to participate in active mobilization due to deep sedation. NMES externally induces muscle contractions in patients who are unable to induce muscle contractions themselves, thereby offering a potential preventive strategy against physical sequelae acquired during the critical illness [32, 53].

## Limitations

Importantly, this review intentionally focused on the description of applied NMES protocols rather than on the mechanistic rationale or primary endpoints of individual studies, in order to capture the full spectrum of stimulation strategies currently used in ICU practice. The main limitation of this review is the incomplete and inconsistent reporting of NMES parameters across the included studies, which reduces the level of detail available for analysis. Additionally, significant heterogeneity in study designs, patient populations, and control conditions greatly limits comparability and hinders a formal meta-analysis. Finally, many of the included trials were small or pilot studies, which limits the external validity and generalizability of the findings.

## Conclusion

The findings of this systematic review highlight the lack of standardized NMES protocols for early rehabilitation in critically ill patients. The wide variation in stimulation parameters and their reporting currently hinders the development of a standardized, evidence-based treatment protocol. We suggest a practical framework for future testing in trials, following published reporting standards. Facilitating comparability across studies with standardized protocols and reporting will further strengthen evidence-based recommendations for NMES use. Based on the available evidence, NMES appears to be a feasible and generally safe adjunct to early rehabilitation in selected critically ill patients, particularly when active mobilization is not possible. However, its routine application in the ICU should currently be guided by individual patient characteristics, careful protocol selection, and realistic expectations regarding clinical benefit until more robust, standardized evidence becomes available.

## Supplementary Information

Additional file 1.

## Data Availability

Additional data are available from the corresponding author upon reasonable request.

## Abbreviations

ICU: Intensive care unit
MRC: Medical research council
NA: Not available
NMES: Neuromuscular electrical stimulation
OBS: Observational study
OSF: Open science framework
PICOS: Participants, Interventions, Comparisons, Outcomes, and Study Design
PRISMA: Preferred reporting items for systematic reviews
RCT: Randomized controlled trial
