# Supplementary material for: Neuromuscular electrical stimulation for early rehabilitation in critically ill patients: a systematic review of applied protocols
Source: Crit Care. 2026 Feb 10;30:92. doi: 10.1186/s13054-026-05873-6 (PMC12930722; doi:10.1186/s13054-026-05873-6)
Supplement: Supplementary file 1 — Additional file 1. [file 13054_2026_5873_MOESM1_ESM.pdf]

Supplement

Neuromuscular electrical stimulation for early mobilization in critically ill patients: a systematic review of applied protocols

Short Title: NMESinICU

Nils Daum[1,2], Nils Drewniok[1], Annika Bald[1], Laura Homann[1], Linus Warner[1], Flora T. Scheffenbichler[3], Antonia Leder[1], Max Liebl[5], Anett Reißhauer[5], Tobias Wollersheim[1], Stefan J. Schaller[1,4], Steffen Weber-Carstens[1], Julius J. Grunow[1]

1 Charité – Universitätsmedizin Berlin, Corporate Member of Freie Universität Berlin and Humboldt Universität zu Berlin, Department of Anesthesiology and Intensive Care Medicine (CCM/CVK), Berlin, Germany

2 Charité – Universitätsmedizin Berlin, Corporate Member of Freie Universität Berlin and Humboldt Universität zu Berlin, Institute of Medical Informatics, Berlin, Germany

3 Ulm University, Department of Anesthesiology and Intensive Care Medicine, Ulm, Germany.

4 Medical University of Vienna, Department of Anaesthesia, Intensive Care Medicine and Pain Medicine, Clinical Division of General Anaesthesia and Intensive Care Medicine, Vienna, Austria

5 Charité – Universitätsmedizin Berlin, Corporate Member of Freie Universität Berlin and Humboldt Universität zu Berlin, Division of Physical Medicine, Berlin, Germany

Table of Contents

Table A1. 3
Table A2. 7
Table A3. 9
Table A4.......... 10
Table A5.......... 11
References.......... 12

Table A1. Systematic search strategy for the databases used in each case.

**Medline and Cochrane Library**	**Search #1 (Positioning)**	(„positioning“ [Title/Abstract] OR „prone position“ [Title/Abstract] OR „prone positioning“ [Title/Abstract] OR „pronation“ [Title/Abstract] OR „proning“ [Title/Abstract] OR „prone“ [Title/Abstract] OR „self-proning“ [Title/Abstract] OR „conscious proning“ [Title/Abstract] OR „conscious prone positioning“ [Title/Abstract] OR „proning in non-intubated“ [Title/Abstract] OR „PINI“ [Title/Abstract] OR „prone ventilation“ [Title/Abstract] OR „face down“ [Title/Abstract] OR „ventral position“ [Title/Abstract] OR „awake proning“ [Title/Abstract] OR „supine position“ [Title/Abstract] OR „supine positioning“ [Title/Abstract] OR „supination“ [Title/Abstract] OR „supine“ [Title/Abstract] OR „lateral position“ [Title/Abstract] OR „lateral positioning“ [Title/Abstract] OR „sitting position“ [Title/Abstract] OR „semisitting position“ [Title/Abstract] OR „horizontal position“ [Title/Abstract] OR „horizontal positioning“ [Title/Abstract] OR „semi-recumbent position“ [Title/Abstract] OR „rotation“ [Title/Abstract] OR „body position“ [Title/Abstract] OR „body positioning“ [Title/Abstract] OR „patient positioning“ [Title/Abstract] OR „positioning therapy“ [Title/Abstract] OR „kinetic therapy“ [Title/Abstract] OR „continuous lateral rotation“ [Title/Abstract] OR „continuous passive motion therapy“ [Title/Abstract] OR „backrest elevation“ [Title/Abstract] OR „axial position change“ [Title/Abstract] OR „body position change“ [Title/Abstract] OR „facedown position“ [Title/Abstract] OR „side position“ [Title/Abstract] OR „posture“ [Title/Abstract] OR „thorax angulation“ [Title/Abstract] OR „kinetics“<br>OR<br>"prone position"[MeSH Terms] OR "pronation"[MeSH Terms] OR "supine position"[MeSH Terms] OR "supination"[MeSH Terms] OR "sitting position"[MeSH Terms] OR "rotation"[MeSH Terms] OR "patient positioning"[MeSH Terms] OR "posture"[MeSH Terms] OR "kinetics"[MeSH Terms])
	**Search #2 (Mobilization)**	(„mobilisation“ [Title/Abstract] OR „mobilization“ [Title/Abstract] OR „early mobilisation“ [Title/Abstract] OR „early mobilization“ [Title/Abstract] OR „early goal-directed mobilization“ [Title/Abstract] OR „early ambulation“ [Title/Abstract] OR „early exercise“ [Title/Abstract] OR „early activity“ [Title/Abstract] OR „early activities“ [Title/Abstract] OR „early goal-directed therapy“ [Title/Abstract] OR „early goal-directed therapies“ [Title/Abstract] OR „accelerated ambulation“ [Title/Abstract] OR „occupational therapy“ [Title/Abstract] OR „occupational therapies“ [Title/Abstract] OR „occupational therapist“ [Title/Abstract] OR „physiotherapy“ [Title/Abstract] OR „physical therapy“ [Title/Abstract] OR „physical therapies“ [Title/Abstract] OR „physical therapist“ [Title/Abstract] OR „physical therapy modality“ [Title/Abstract] OR „physical therapy modalities“ [Title/Abstract] OR „physical fitness“ [Title/Abstract] OR „mobility therapy“ [Title/Abstract] OR „exercise therapy“ [Title/Abstract] OR „exercise therapies“ [Title/Abstract] OR „movement therapy“ [Title/Abstract] OR „movement therapies“ [Title/Abstract] OR „myofunctional therapy“ [Title/Abstract] OR „myofunctional therapies“ [Title/Abstract] OR „kinesiotherapy“ [Title/Abstract] OR „motion therapy“ [Title/Abstract] OR „mobility intervention“ [Title/Abstract] OR „motor activity“ [Title/Abstract] OR „motor activities“ [Title/Abstract] OR „rehabilitation“ [Title/Abstract] OR „muscle training“ [Title/Abstract] OR „muscle strength“ [Title/Abstract] OR „muscle strengthening“ [Title/Abstract] OR „musculoskeletal manipulations“ [Title/Abstract] OR „resistance training“ [Title/Abstract] OR „strength training“ [Title/Abstract] OR „endurance training“ [Title/Abstract] OR „fitness training“ [Title/Abstract] OR „high-intensity interval training“ [Title/Abstract] OR „physical conditioning“ [Title/Abstract] OR „physical medicine“ [Title/Abstract] OR „physical endurance“ [Title/Abstract] OR „physical strength“ [Title/Abstract] OR „walking“ [Title/Abstract] OR „exercise“ [Title/Abstract] OR „exercises“ [Title/Abstract] OR „exercising“ [Title/Abstract] OR „exercise tolerance“ [Title/Abstract] OR „functional status“ [Title/Abstract] OR „load bearing“ [Title/Abstract] OR „loadbearing“ [Title/Abstract] OR „weight bearing“ [Title/Abstract] OR „weightbearing“ [Title/Abstract] OR „locomotion“ [Title/Abstract] OR „locomotor activity“ [Title/Abstract] OR „locomotor activities“ [Title/Abstract] OR „muscle strength“ [Title/Abstract] OR „muscle strengthening“ [Title/Abstract] OR „recovery of

		function“ [Title/Abstract] OR „function recovery“ [Title/Abstract] OR „functional recoveries“ [Title/Abstract] OR „functional recovery“ [Title/Abstract] OR „functional training“ [Title/Abstract] OR „joint mobilization“ [Title/Abstract] OR „joint mobilization“ [Title/Abstract] OR „movement“ [Title/Abstract] OR „physical stimulation“ [Title/Abstract] OR „stimulation“ [Title/Abstract] OR „cycle“ [Title/Abstract] OR „cycling“ [Title/Abstract] OR „leg-cycle“ [Title/Abstract] OR „leg-cycling“ [Title/Abstract] OR „bicycle“ [Title/Abstract] OR „bicycling“ [Title/Abstract] OR „in-bed cycling“ [Title/Abstract] OR „ergometer“ [Title/Abstract] OR „ergometry“ [Title/Abstract] OR „electrostimulation“ [Title/Abstract] OR „electric stimulation“ [Title/Abstract] OR „electrical stimulation“ [Title/Abstract] OR „electrotherapy“ [Title/Abstract] OR „electrotherapies“ [Title/Abstract] OR „electric stimulation therapy“ [Title/Abstract] OR „electrical stimulation therapy“ [Title/Abstract] OR „electrical stimulation therapies“ [Title/Abstract] OR „neuromuscular stimulation“ [Title/Abstract] OR „neuromuscular electric stimulation“ [Title/Abstract] OR „neuromuscular electrical stimulation“ [Title/Abstract] OR „transcutaneous electric stimulation“ [Title/Abstract] OR „transcutaneous electrical stimulation“ [Title/Abstract] OR „muscle stimulation“ [Title/Abstract] OR „muscular stimulation“ [Title/Abstract] OR „muscle excitation“ [Title/Abstract] OR „electromyostimulation“ [Title/Abstract] OR „functional electrical stimulation“ [Title/Abstract] OR „TENS“ [Title/Abstract] OR „NMES“ [Title/Abstract] OR „EMS“ [Title/Abstract] OR „FES“ [Title/Abstract] OR „assistive technology devices“ [Title/Abstract]<br>OR<br>"early ambulation"[MeSH Terms] OR "early goal-directed therapy"[MeSH Terms] OR "occupational therapy"[MeSH Terms] OR "physical therapy modalities"[MeSH Terms] OR "physical fitness"[MeSH Terms] OR "exercise therapy"[MeSH Terms] OR "myofunctional therapy"[MeSH Terms] OR "motor activity"[MeSH Terms] OR "rehabilitation"[MeSH Terms] OR "muscle strength"[MeSH Terms] OR "musculoskeletal manipulations"[MeSH Terms] OR "resistance training"[MeSH Terms] OR "endurance training"[MeSH Terms] OR "high-intensity interval training"[MeSH Terms] OR "physical endurance"[MeSH Terms] OR "walking"[MeSH Terms] OR "exercise"[MeSH Terms] OR "exercise tolerance"[MeSH Terms] OR "functional status"[MeSH Terms] OR "weight bearing"[MeSH Terms] OR "locomotion"[MeSH Terms] OR "muscle strength"[MeSH Terms] OR "recovery of function"[MeSH Terms] OR "movement"[MeSH Terms] OR "physical stimulation"[MeSH Terms] OR "bicycling"[MeSH Terms] OR "ergometry"[MeSH Terms] OR "electric stimulation"[MeSH Terms] OR "electric stimulation therapy"[MeSH Terms])
	**Search #3 (Critical Care)**	(„critically ill“ [Title/Abstract] OR „critical illness“ [Title/Abstract] OR “catastrophic illness“ [Title/Abstract] OR „critical care“ [Title/Abstract] OR „critical care unit“ [Title/Abstract] OR „critical care units“ [Title/Abstract] OR „intensive care“ [Title/Abstract] OR „intensive care unit“ [Title/Abstract] OR „intensive care units“ [Title/Abstract] OR „respiratory care unit“ [Title/Abstract] OR „respiratory care units“ [Title/Abstract] OR „intensive treatment unit“ [Title/Abstract] OR „intensive therapy unit“ [Title/Abstract] OR „special care unit“ [Title/Abstract] OR „ICU“ [Title/Abstract] OR „ITU“ [Title/Abstract] OR „MICU“ [Title/Abstract] OR „controlled ventilation“ [Title/Abstract] OR „intensive care patient“ [Title/Abstract] OR „invasive ventilation“ [Title/Abstract] OR „invasive respiration“ [Title/Abstract] OR „artificial respiration“ [Title/Abstract] OR „mechanical ventilation“[Title/Abstract]<br>OR<br>"critical illness"[MeSH Terms] OR "catastrophic illness"[MeSH Terms] OR "critical care"[MeSH Terms] OR "intensive care units"[MeSH Terms] OR "respiratory care units"[MeSH Terms])
	**Final Search (#1 OR #2) AND #3**	
**Pedro**		Abstract & Title: mob* critical*
		Abstract & Title: mob* ICU
		Abstract & Title: mob* intensive

		Abstract & Title: pron* critical*
		Abstract & Title: pron* ICU
		Abstract & Title: pron* intensive
		Abstract & Title: neuromusc* critical*
		Abstract & Title: neuromusc* ICU
		Abstract & Title: neuromusc* intensive
**Cinahl**	**Search #1 (Positioning)**	„positioning“ OR „prone position“ OR „prone positioning“ OR „pronation“ OR „proning“ OR „prone“ OR „self-proning“ OR „conscious proning“ OR „conscious prone positioning“ OR „proning in non-intubated“ OR „PINI“ OR „prone ventilation“ OR „face down“ OR „ventral position“ OR „awake proning“ OR „supine position“ OR „supine positioning“ OR „supination“ OR „supine“ OR „lateral position“ OR „lateral positioning“ OR „sitting position“ OR „semisitting position“ OR „horizontal position“ OR „horizontal positioning“ OR „semi-recumbent position“ OR „rotation“ OR „body position“ OR „body positioning“ OR „patient positioning“ OR „positioning therapy“ OR „kinetic therapy“ OR „continuous lateral rotation“ OR „continuous passive motion therapy“ OR „backrest elevation“ OR „axial position change“ OR „body position change“ OR „facedown position“ OR „side position“ OR „posture“ OR „thorax angulation“ OR „kinetics“
	**Search #2 (Mobilization)**	„mobilisation“ OR „mobilization“ OR „early mobilisation“ OR „early mobilization“ OR „early goal-directed mobilisation“ OR „early goal-directed mobilization“ OR „early ambulation“ OR „early exercise“ OR „early activity“ OR „early activities“ OR „early goal-directed therapy“ OR „early goal-directed therapies“ OR „accelerated ambulation“ OR „occupational therapy“ OR „occupational therapies“ OR „occupational therapist“ OR „physiotherapy“ OR „physical therapy“ OR „physical therapies“ OR „physical therapist“ OR „physical therapy modality“ OR „physical therapy modalities“ OR „physical fitness“ OR „mobility therapy“ OR „mobility therapies“ OR „exercise therapy“ OR „exercise therapies“ OR „movement therapy“ OR „movement therapies“ OR „myofunctional therapy“ OR „myofunctional therapies“ OR „kinesiotherapy“ OR „kinesiotherapies“ OR „motion therapy“ OR „mobility intervention“ OR „motor activity“ OR „motor activities“ OR „rehabilitation“ OR „muscle training“ OR „muscle strength“ OR „muscle strengthening“ OR „musculoskeletal manipulations“ OR „resistance training“ OR „strength training“ OR „endurance training“ OR „fitness training“ OR „high-intensity interval training“ OR „physical conditioning“ OR „physical medicine“ OR „physical endurance“ OR „physical strength“ OR „walking“ OR „exercise“ OR „exercises“ OR „exercising“ OR „exercise tolerance“ OR „functional status“ OR „load bearing“ OR „loadbearing“ OR „weight bearing“ OR „weightbearing“ OR „locomotion“ OR „locomotor activity“ OR „locomotor activities“ OR „muscle strength“ OR „muscle strengthening“ OR „recovery of function“ OR „function recovery“ OR „functional recoveries“ OR „functional recovery“ OR „functional training“ OR „joint mobilization“ OR „joint mobilization“ OR „movement“ OR „physical stimulation“ OR „stimulation“ OR „cycle“ OR „cycling“ OR „leg-cycle“ OR „leg-cycling“ OR „bicycle“ OR „bicycling“ OR „in-bed cycling“ OR „ergometer“ OR „ergometry“ OR „electrostimulation“ OR „electric stimulation“ OR „electrical stimulation“ OR „electrotherapy“ OR „electrotherapies“ OR „electric stimulation therapy“ OR „electric stimulation therapies“ OR „electrical stimulation therapy“ OR „electrical stimulation therapies“ OR „neuromuscular stimulation“ OR „neuromuscular electric stimulation“ OR „neuromuscular electrical stimulation“ OR „transcutaneous electric stimulation“ OR „transcutaneous electrical stimulation“ OR „muscle stimulation“ OR „muscular stimulation“ OR „muscle excitation“ OR „electromyostimulation“ OR „functional electrical stimulation“ OR „TENS“ OR „NMES“ OR „EMS“ OR „FES“ OR „assistive technology devices“

**Search #3 (Critical Care)**	„critically ill“ OR „critical illness“ OR “catastrophic illness“ OR „critical care“ OR „critical care unit“ OR „critical care units“ OR „intensive care“ OR „intensive care unit“ OR „intensive care units“ OR „respiratory care unit“ OR „respiratory care units“ OR „intensive treatment unit“ OR „intensive therapy unit“ OR „special care unit“ OR „ICU“ OR „ITU“ OR „MICU“ OR „controlled ventilation“ OR „intensive care patient“ OR „invasive ventilation“ OR „invasive respiration“ OR „artificial respiration“ OR „mechanical ventilation“
**Final Search (#1 OR #2) AND #3**	

Table A2. Patient characteristics of studies with one intervention. Numbers are shown with percentages, means and standard deviations are shown in round brackets and interquartile range ranges with 25th and 75th percentile in squared brackets.

Author	Year	Patients intervention group 1 (n)	Age intervention group 1 (years)	Sex intervention group 1 (male, n, %)	APACHE II score intervention 1	SOFA score intervention 1	Main admission disease intervention 1	Patients control group (n)	Age control group (years)	Sex control group (male, n, %)	APACHE II score control	SOFA score control group	Main admission disease control group
Abu-Khaber [1]	2013	50	59.1 (5.3)	24 (60%)	24.5 (6.8)	na	Respiratory	40	57.6 (6.8)	27 (68%)	26.1 (5.3)	na	Respiratory
Baron [2]	2022	76	82.6 (17,4)	38 (50%)	na	2.0 [0.0-4.0]	Stroke	73	63.7 (18.2)	42 (58%)	na	2.0 [0.0-4.0]	Stroke
Berney [3]	2021	80	61.0 [51.0-69.0]	53 (66%)	22.0 [16.0-27.0]	11.0 [7.0-13.0]	Respiratory	82	59.0 [48.0-67.0]	54 (66%)	23.0 [17.0-27.0]	10.0 [7.0-14.0]	Respiratory
Campos [4]	2022	34	42.5 (14.9)	24 (70%)	na	8.8 (3.0)	Surgical	40	46.7 (17.9)	26 (65%)	na	9.4 (2.7)	Surgical
Cerqueira [5]	2018	26	41.8 (13.2)	18 (69%)	na	na	Surgical	33	42.2 (14.4)	23 (70%)	na	na	Surgical
Dall'Acqua [6]	2017	11	56.0 (13.0)	7 (64%)	26.0 (5.0)	na	Sepsis	14	61.0 (15.0)	9 (64%)	29.0 (7.0)	na	Sepsis
Dirks [7]	2015	6	63.3 (6.0)	3 (50%)	29.3 (2.0)	na	Cerebral						
Falavigna [8]	2013	11	34.0 (17.3)	na	15.7 (4.5)	na	Sepsis						
Figueiredo [9]	2023	29	65.0 [54.5–78.5]	9 (31%)	na	na	Resuscitati on	20	64.5 [51.0–69.8]	9 (45%)	na	na	None
Figueiredo [10]	2024	20	61.0 (20.0)	13 (65%)	na	na	Arrythmia						
Fischer [11]	2016	27	63.0 (15.5)	18 (67%)	na	9.0 [1.0-15.0]	Surgical	27	69.7 (13.1)	20 (74%)	na	7.0 [1.0-11.0]	Surgical
Fossat [12]	2018	158	63.0 (13.0)	103 (65%)	na	9.0 [6.0-12.0]	Respiratory	154	66.0 (15.0)	98 (64%)	na	8.5 [6.0-12.0]	Respiratory
Gerovasili [13]	2009	24	59.0 (23.0)	6 (46%)	19.0 (3.0)	10.0 (3.0)	Sepsis	25	56.0 (19.0)	8 (62%)	18.0 (6.0)	8.0 (3.0)	Sepsis
Gruther [14]	2010	8	52.0 (10.0)	7 (88%)	na	na	Trauma	9	48.0 (12.0)	8 (89%)	na	na	Trauma
Hirose [15]	2013	9	49.9 (16.5)	8 (89%)	na	na	Cerebral	6	59.8 (17.2)	3 (50%)	na	na	Cerebral
Karatzanos [16]	2012	24	55.0 (20.0)	19 (79%)	16.0 (4.0)	8.0 (3.0)	Cerebral	28	59.0 (21.0)	22 (79%)	19.0 (5.0)	8.0 (3.0)	Sepsis
Kho [17]	2015	16	54.0 (16.0)	7 (44%)	25.0 (8.0)	6.2 (4.7)	Sepsis	18	56.0 (18.0)	10 (56%)	25.0 (6.0)	5.6 (1.6)	Sepsis

Kourek [18]	2024	16	46.6 (13.7)	6 (37%)	na	na	Cardiac						
Koutsioumpa [19]	2018	38	64.0 (12.4)	26 (68%)	17.0 (7.6)	8.0 (4.2)	Respiratory	42	66.0 (13.1)	34 (81%)	21.0 (7.9)	7.0 (4.1)	Respiratory
Leite [20]	2018	24	48.8 (19.7)	18 (75%)	18.7 (4.0)	na	Cerebral	26	42.4 (12.7)	20 (77%)	18.9 (1.9)	na	Cerebral
Liu [21]	2023	40	58.1 (15.5)	25 (63%)	20.6 (6.4)	na	Sepsis	40	59.1 (16.0)	23 (58%)	19.8 (6.4)	na	Sepsis
Mahran [22]	2023	60	31.0 (10.0)	46 (77%)	12.3 (4.2)	na	Cerebral	58	32.0 (9.0)	50 (86%)	15.4 (7.3)	na	Cerebral
Medrinal [23]	2018	19	65.3 (9.7)	13 (68%)	na	na	Sepsis						
Meesen [24]	2010	7	65.3 (16.5)	3 (43%)	na	na	Surgical	12	67.2 (13.2)	9 (75%)	na	na	Surgical
Nakamura [25]	2019	21	76.6 (11.0)	14 (67%)	22.8 (6.2)	8.6 (3.7)	Sepsis	16	74.6 (13.1)	11 (69%)	22.9 (3.9)	8.7 (2.8)	Sepsis
Nakanishi [26]	2020	17	73.0 (3.0)	12 (71%)	25.0 [20.0–31.0]	9.0 [6.0–12.0]	Respiratory	19	66.0 (3.0)	12 (63%)	22.0 [19.0–30.0]	7.0 [5.0–10.0]	Respiratory
Poulsen [27]	2011	8	67.0 [64.0-72.0]	8 (100%)	25.0 [20.0-29.0]	13.0 [11.0-15.0]	Sepsis						
Rodriguez [28]	2012	14	72.0 [63.0-80.0]	7 (50%)	20.0 [18.0-27.0]	10.0 [9.0-12.0]	Respiratory						
Routsi [29]	2010	68	61.0 (19.0)	46 (68%)	18.0 (4.0)	9.0 (3.0)	Cerebral	72	58.0 (18.0)	49 (68%)	18.0 (5.0)	9.0 (3.0)	Cerebral
Segers [30]	2021	47	60.0 (15.0)	25 (53%)	26.0 (8.0)	na	Respiratory						
Silva [31]	2017	11	39.0 (16.0)	11 (100%)	15.1 (0.9)	na	Trauma						
Silva [32]	2019	30	30.0 [27.0-33.0]	26 (87%)	11.0 [8.0–13.0]	5.0 [5.0–8.0]	Cerebral	30	33.0 [29.0-37.0]	26 (87%)	11.0 [9.0–14.0]	6.0 [4.0–9.0]	Cerebral
Stefanou [33]	2016	32	58.0 (14.0)	23 (72%)	21.0 (8.0)	7.0 (3.0)	Surgical						
Verceles [34]	2023	16	62.0 (9.3)	8 (50%)	17.8 (5.5)	na	Respiratory	23	62.0 (9.3)	10 (43%)	15.4 (7.4)	na	Respiratory
Vieira [35]	2023	20	34.7 (11.2)	16 (80%)	16.1 (4.6)	na	Cerebral	20	36.5 (13.5)	16 (80%)	16.7 (4.5)	na	Cerebral
Waldauf [36]	2021	75	59.0 (15.1)	53 (71%)	22.1 (5.2)	8.8 (2.6)	Respiratory	75	62,3 ± 15,4	57 (76%)	22.2 (7.7)	8.8 (3.2)	Respiratory
Wollersheim [37]	2019	33	54.0 [45.0-68.0]	24 (73%)	24.0 [20.0-28.0]	12.0 [11.0-14.0]	Respiratory	17	45 [39-61]	9 (53%)	26.0 [19.0-31.0]	14.0 [12.0-17.0]	Respiratory
Woo [38]	2018	10	63.5 [53.8-71.0]	7 (70%)	23.5 [21.2-29.0]	2.0 [2.0-4.0]	Surgical						
Zulbaran-Rojas [39]	2022	8	66.8 (9.8)	3 (38%)	na	na	Respiratory	8	62.9 (9.5)	6 (75%)	na	na	Respiratory

na not available

Table A3. Patient characteristics of studies with two or more interventions. Numbers are shown with percentages, means and standard deviations are shown in round brackets and interquartile range ranges with 25th and 75th percentile in squared brackets.

Author	Year	Patients intervention group 1 (n)	Age intervention group 1 (years)	Sex intervention group 1 (male, n, %)	APACHE II score intervention 1	SOFA score intervention 1	Main admission disease	Patients intervention group 2 (n)	Age intervention group 2 (years)	Sex intervention group 2 (male, n, %)	APACHE II score intervention 2	SOFA score intervention 2	Main admission disease	Patients intervention group 3 (n)	Age intervention group 3 (years)	Sex intervention group 3 (male, n, %)	APACHE II score intervention 3	SOFA score intervention 3	Main admission disease	Patients control group (n)	Age control group (years)	Sex control group (male, n, %)	APACHE II score control	SOFA score control group	Main admission disease
Akar [40]	2017	10	70.0 (12.3)	4 (40%)	na	na	Respi ratory	10	62.8 (6.8)	6 (60%)	na	na	Respi ratory							10	68.0 (17.8)	5 (50%)	na	na	Respi ratory
Bao [41]	2022	20	52.8 (10,7)	14 (70%)	9.4 (4.5)	na	Trauma	20	51.1 (17.6)	18 (90%)	9.7 (5.2)	na	Trauma							20	52.5 (12.5)	19 (95%)	9.2 (3.0)	na	Trauma
Dos Santos [42]	2020	12	55.6 (10.8)	8 (67%)	15.5 (3.5)	na	Respi ratory	13	55.3 (12.7)	9 (69%)	17.0 (3.8)	na	Respi ratory	11	50.2 (12.8)	7 (67%)	6.1 (2.9)	na	Respi ratory	15	51.8 (12.8)	11 (73%)	15.3 (3.7)	na	Sepsis
Guerra-Vega [43]	2025	18	62.0 (11.0)	12 (67%)	33.0 (3.0)	14.0 (1.0)	Sepsis	17	59.0 (16.0)	9 (53%)	31.0 (3.0)	15.0 (2.0)	Sepsis							17	53.0 (18.0)	8 (47%)	31.0 (2.0)	14.0 (1.0)	Sepsis
Othman [44]	2023	30	38.8 (11.0)	16 (53%)	29.9 (3.9)	na	Respi ratory	30	40.3 (9.3)	15 (50%)	28.0 (4.4)	na	Respi ratory	30	45.6 (8.9)	16 (53%)	29.8 (3.3)	na	Respi ratory	30	42.4 (13.2)	14 (47%)	28.4 (4.0)	na	Respi ratory

na not available

Table A4. Risk of Bias Assessment using the ROBINS-E Tool for Prospective Non-randomized Studies.

Author	Year	D1	D2	D3	D4	D5	D6	D7	Overall
Figueiredo	2023	High	Some concerns	Low	Some concerns	Low	Some concerns	Some concerns	**High**
Figueiredo	2024	High	High	Low	Low	Low	Low	Some concerns	**High**
Kourek	2024	Some concerns	High	Low	Low	Low	Some concerns	Some concerns	**High**
Silva	2017	High	High	Some concerns	Some concerns	Low	Some concerns	Low	**High**

D1 Confounding; D2 Selection of participants; D3 Classification of exposure; D4 Deviations from intended exposure; D5 Missing data; D6 Measurement of outcomes; D7 Selection of the reported result

Table A5. Risk of Bias Assessment using the RoB-2 Tool for Randomized Controlled Studies.

Author	D1	D2	D3	D4	D5	Overall
Abu-Khaber	-	-	!	-	!	-
Akar	!	+	+	+	+	!
Bao	+	+	+	!	+	!
Baron	+	!	!	+	!	!
Berney	+	+	+	!	+	!
Campos	+	+	!	!	+	!
Cerqueira	+	!	-	+	!	-
Dall'Acqua	+	+	+	+	!	!
Dirks	+	!	-	+	!	-
Dos Santos	+	-	-	+	!	-
Falavigna	!	+	+	+	!	!
Fischer	!	+	+	+	!	!
Fossat	+	+	+	+	+	+
Gerovasili	+	+	+	+	+	+
Gruther	+	+	!	+	!	!
Hirose	-	+	+	-	!	-
Karatzanos	+	+	!	!	+	!
Kho	+	+	!	+	-	-
Koutsioumpa	!	+	-	!	!	-
Leite	-	+	-	+	-	-
Liu	!	-	+	-	!	-
Mahran	!	-	+	+	-	-
Medrinal	+	+	+	!	!	!
Meesen	+	+	!	+	!	!
Nakamura	!	+	+	+	!	!
Nakanishi	+	+	+	!	!	!
Othman	+	!	-	!	-	-
Poulsen	+	+	+	+	!	!
Rodriguez	+	+	+	+	!	!
Routsi	+	+	+	+	+	+
Segers	+	+	+	!	!	!
Silva	+	!	+	!	+	!
Stefanou	!	!	+	!	!	!
Verceles	+	+	+	+	!	!
Vieira	+	!	!	!	!	!
Waldauf	+	+	+	!	+	!
Wollersheim	+	+	!	+	!	!
Woo	-	!	+	!	-	-
Zulbaran-Rojas	!	!	!	-	!	!

+ Low risk
! Some concerns
- High risk

D1 Randomisation process
D2 Deviations from the intended interventions
D3 Missing outcome data
D4 Measurement of the outcome
D5 Selection of the reported result

References

1. Abu-Khaber HA, Abouelela AMZ, Abdelkarim EM: **Effect of electrical muscle stimulation on prevention of ICU acquired muscle weakness and facilitating weaning from mechanical ventilation**. *Alexandria Journal of Medicine* 2013, **49**(4):309-315.
2. Baron MV, Silva PE, Koepp J, Urbanetto JS, Santamaria AFM, Dos Santos MP, de Mello Pinto MV, Brandenburg C, Reinheimer IC, Carvalho S *et al*: **Efficacy and safety of neuromuscular electrical stimulation in the prevention of pressure injuries in critically ill patients: a randomized controlled trial**. *Ann Intensive Care* 2022, **12**(1):53.
3. Berney S, Hopkins RO, Rose JW, Koopman R, Puthucheary Z, Pastva A, Gordon I, Colantuoni E, Parry SM, Needham DM *et al*: **Functional electrical stimulation in-bed cycle ergometry in mechanically ventilated patients: a multicentre randomised controlled trial**. *Thorax* 2021, **76**(7):656-663.
4. Campos DR, Bueno TBC, Anjos J, Zoppi D, Dantas BG, Gosselink R, Guirro RRJ, Borges MC: **Early Neuromuscular Electrical Stimulation in Addition to Early Mobilization Improves Functional Status and Decreases Hospitalization Days of Critically Ill Patients**. *Crit Care Med* 2022, **50**(7):1116-1126.
5. Fontes Cerqueira TC, Cerqueira Neto ML, Cacau LAP, Oliveira GU, Silva Junior WMD, Carvalho VO, Mendonca JT, Santana Filho VJ: **Ambulation capacity and functional outcome in patients undergoing neuromuscular electrical stimulation after cardiac valve surgery: A randomised clinical trial**. *Medicine (Baltimore)* 2018, **97**(46):e13012.
6. Dall' Acqua AM, Sachetti A, Santos LJ, Lemos FA, Bianchi T, Naue WS, Dias AS, Sbruzzi G, Vieira SR: **Use of neuromuscular electrical stimulation to preserve the thickness of abdominal and chest muscles of critically ill patients: A randomized clinical trial**. *J Rehabil Med* 2017, **49**(1):40-48.
7. Dirks ML, Hansen D, Van Assche A, Dendale P, Van Loon LJ: **Neuromuscular electrical stimulation prevents muscle wasting in critically ill comatose patients**. *Clin Sci (Lond)* 2015, **128**(6):357-365.
8. Falavigna LF, Silva MG, De Almeida Freitas AL, Silva PFDS, Paiva Júnior MDS, De Castro CMMB, Andrade MDA, Cavalcanti Gallindo MA, Ribeiro LC, Ramos FF *et al*: **Effects of electrical muscle stimulation early in the quadriceps and tibialis anterior muscle of critically ill patients**. *Physiotherapy Theory and Practice* 2014, **30**(4):223-228.
9. de Gomes Figueiredo T, Frazão M, Werlang LA, Peltz M, Sobral Filho DC: **Functional electrical stimulation cycling-based muscular evaluation method in mechanically ventilated patients**. *Artif Organs* 2024, **48**(3):254-262.
10. Figueiredo T, Frazão M, Werlang LA, Kunz A, Peltz M, Furtado VC, Júnior EB, Júnior JM, Silva RM, Sobral Filho DC: **Safety and feasibility of a functional electrical stimulation cycling-based muscular dysfunction diagnostic method in mechanically ventilated patients**. *Artif Organs* 2024, **48**(7):713-722.
11. Fischer A, Spiegl M, Altmann K, Winkler A, Salamon A, Themessl-Huber M, Mouhieddine M, Strasser EM, Schiferer A, Paternostro-Sluga T *et al*: **Muscle mass, strength and functional outcomes in critically ill patients after cardiothoracic surgery: does neuromuscular electrical stimulation help? The Catastim 2 randomized controlled trial**. *Crit Care* 2016, **20**:30.

12. Fossat G, Baudin F, Courtes L, Bobet S, Dupont A, Bretagnol A, Benzekri-Lefèvre D, Kamel T, Muller G, Bercault N *et al*: **Effect of In-Bed Leg Cycling and Electrical Stimulation of the Quadriceps on Global Muscle Strength in Critically Ill Adults: A Randomized Clinical Trial**. *Jama* 2018, **320**(4):368-378.
13. Gerovasili V, Stefanidis K, Vitzilaios K, Karatzanos E, Politis P, Koroneos A, Chatzimichail A, Routsi C, Roussos C, Nanas S: **Electrical muscle stimulation preserves the muscle mass of critically ill patients: a randomized study**. *Crit Care* 2009, **13**(5):R161.
14. Gruther W, Kainberger F, Fialka-Moser V, Paternostro-Sluga T, Quittan M, Spiss C, Crevenna R: **Effects of neuromuscular electrical stimulation on muscle layer thickness of knee extensor muscles in intensive care unit patients: a pilot study**. *J Rehabil Med* 2010, **42**(6):593-597.
15. Hirose T, Shiozaki T, Shimizu K, Mouri T, Noguchi K, Ohnishi M, Shimazu T: **The effect of electrical muscle stimulation on the prevention of disuse muscle atrophy in patients with consciousness disturbance in the intensive care unit**. *J Crit Care* 2013, **28**(4):536.e531-537.
16. Karatzanos E, Gerovasili V, Zervakis D, Tripodaki ES, Apostolou K, Vasileiadis I, Papadopoulos E, Mitsiou G, Tsimpouki D, Routsi C *et al*: **Electrical muscle stimulation: an effective form of exercise and early mobilization to preserve muscle strength in critically ill patients**. *Crit Care Res Pract* 2012, **2012**:432752.
17. Kho ME, Truong AD, Zanni JM, Ciesla ND, Brower RG, Palmer JB, Needham DM: **Neuromuscular electrical stimulation in mechanically ventilated patients: A randomized, sham-controlled pilot trial with blinded outcome assessment**. *Journal of Critical Care* 2015, **30**(1):32-39.
18. Kourek C, Raidou V, Antonopoulos M, Dimopoulou M, Koliopoulou A, Karatzanos E, Pitsolis T, Ieromonachos K, Nanas S, Adamopoulos S *et al*: **Safety and Feasibility of Neuromuscular Electrical Stimulation in Patients with Extracorporeal Membrane Oxygenation**. *J Clin Med* 2024, **13**(13).
19. Koutsioumpa E, Makris D, Theochari A, Bagka D, Stathakis S, Manoulakas E, Sgantzos M, Zakynthinos E: **Effect of transcutaneous electrical neuromuscular stimulation on myopathy in intensive care patients**. *American Journal of Critical Care 2018 Nov;27(6):495-503* 2018.
20. Leite MA, Osaku EF, Albert J, Costa C, Garcia AM, Czapiesvski FDN, Ogasawara SM, Bertolini GRF, Jorge AC, Duarte PAD: **Effects of neuromuscular electrical stimulation of the quadriceps and diaphragm in critically ill patients: a pilot study**. *Critical Care Research and Practice 2018 Jul 8;(4298583):Epub* 2018.
21. Liu Y, Gong Y, Zhang C, Meng P, Gai Y, Han X, Yuan Z, Xing J, Dong Z: **Effect of neuromuscular electrical stimulation combined with early rehabilitation therapy on mechanically ventilated patients: a prospective randomized controlled study**. *BMC Pulm Med* 2023, **23**(1):272.
22. Mahran GSK, Mehany MM, Abbas MS, Shehata A-E, AbdElhafeez AS, Obiedallah AA, Mohamed SA: **Short-Term Outcomes of Neuromuscular Electrical Stimulation in Critically Ill Patients**. *Critical Care Nursing Quarterly* 2023, **46**(2):126-135.
23. Medrinal C, Combret Y, Prieur G, Robledo Quesada A, Bonnevie T, Gravier FE, Dupuis Lozeron E, Frenoy E, Contal O, Lamia B: **Comparison of exercise intensity during four early rehabilitation techniques in sedated and**

ventilated patients in ICU: a randomised cross-over trial. *Crit Care* 2018, **22**(1):110.
24. Meesen RL, Dendale P, Cuypers K, Berger J, Hermans A, Thijs H, Levin O: **Neuromuscular electrical stimulation as a possible means to prevent muscle tissue wasting in artificially ventilated and sedated patients in the intensive care unit: A pilot study**. *Neuromodulation* 2010, **13**(4):315-320; discussion 321.
25. Nakamura K, Kihata A, Naraba H, Kanda N, Takahashi Y, Sonoo T, Hashimoto H, Morimura N: **Efficacy of belt electrode skeletal muscle electrical stimulation on reducing the rate of muscle volume loss in critically ill patients: A randomized controlled trial**. *J Rehabil Med* 2019, **51**(9):705-711.
26. Nakanishi N, Oto J, Tsutsumi R, Yamamoto T, Ueno Y, Nakataki E, Itagaki T, Sakaue H, Nishimura M: **Effect of Electrical Muscle Stimulation on Upper and Lower Limb Muscles in Critically Ill Patients: A Two-Center Randomized Controlled Trial**. *Crit Care Med* 2020, **48**(11):e997-e1003.
27. Poulsen JB, Møller K, Jensen CV, Weisdorf S, Kehlet H, Perner A: **Effect of transcutaneous electrical muscle stimulation on muscle volume in patients with septic shock**. *Crit Care Med* 2011, **39**(3):456-461.
28. Rodriguez PO, Setten M, Maskin LP, Bonelli I, Vidomlansky SR, Attie S, Frosiani SL, Kozima S, Valentini R: **Muscle weakness in septic patients requiring mechanical ventilation: protective effect of transcutaneous neuromuscular electrical stimulation**. *J Crit Care* 2012, **27**(3):319.e311-318.
29. Routsi C, Gerovasili V, Vasileiadis I, Karatzanos E, Pitsolis T, Tripodaki E, Markaki V, Zervakis D, Nanas S: **Electrical muscle stimulation prevents critical illness polyneuromyopathy: a randomized parallel intervention trial**. *Crit Care* 2010, **14**(2):R74.
30. Segers J, Vanhorebeek I, Langer D, Charususin N, Wei W, Frickx B, Demeyere I, Clerckx B, Casaer M, Derese I *et al*: **Early neuromuscular electrical stimulation reduces the loss of muscle mass in critically ill patients -- a within subject randomized controlled trial [with consumer summary]**. *Journal of Critical Care 2021 Apr;62:65-71* 2021.
31. Silva PE, Babault N, Mazullo JB, De Oliveira TP, Lemos BL, Carvalho VO, Durigan JLQ: **Safety and feasibility of a neuromuscular electrical stimulation chronaxie-based protocol in critical ill patients: A prospective observational study**. *Journal of Critical Care* 2017, **37**:141-148.
32. Silva PE, De Cassia Marqueti R, Livino-De-Carvalho K, De Araujo AET, Castro J, Da Silva VM, Vieira L, Souza VC, Dantas LO, Cipriano G *et al*: **Neuromuscular electrical stimulation in critically ill traumatic brain injury patients attenuates muscle atrophy, neurophysiological disorders, and weakness: A randomized controlled trial**. *Journal of Intensive Care* 2019, **7**(1):59.
33. Stefanou C, Karatzanos E, Mitsiou G, Psarra K, Angelopoulos E, Dimopoulos S, Gerovasili V, Boviatsis E, Routsi C, Nanas S: **Neuromuscular electrical stimulation acutely mobilizes endothelial progenitor cells in critically ill patients with sepsis**. *Ann Intensive Care* 2016, **6**(1):21.
34. Verceles AC, Serra M, Davis D, Alon G, Wells CL, Parker E, Sorkin J, Bhatti W, Terrin ML: **Combining exercise, protein supplementation and electric stimulation to mitigate muscle wasting and improve outcomes for survivors of critical illness-The ExPrES study**. *Heart Lung* 2023, **58**:229-235.

35. Vieira L, Silva PE, de Melo PF, Maldaner V, Durigan JQ, Marqueti RC, Nobrega O, Mathur S, Burtin C, Barin F *et al*: **Early Neuromuscular Electrical Stimulation Preserves Muscle Size and Quality and Maintains Systemic Levels of Signaling Mediators of Muscle Growth and Inflammation in Patients with Traumatic Brain Injury: A Randomized Clinical Trial**. *Crit Care Res Pract* 2023, **2023**:9335379.
36. Waldauf P, Hruskova N, Blahutova B, Gojda J, Urban T, Krajcova A, Fric M, Jiroutkova K, Rasova K, Duska F: **Functional electrical stimulation-assisted cycle ergometry-based progressive mobility programme for mechanically ventilated patients: randomised controlled trial with 6 months follow-up [with consumer summary]**. *Thorax 2021 Jul;76(7):664-671* 2021.
37. Wollersheim T, Grunow JJ, Carbon NM, Haas K, Malleike J, Ramme SF, Schneider J, Spies CD, Märdian S, Mai K *et al*: **Muscle wasting and function after muscle activation and early protocol-based physiotherapy: an explorative trial**. *Journal of Cachexia, Sarcopenia and Muscle* 2019, **10**(4):734-747.
38. Woo K, Kim J, Kim HB, Choi H, Kim K, Lee D, Na S: **The Effect of Electrical Muscle Stimulation and In-bed Cycling on Muscle Strength and Mass of Mechanically Ventilated Patients: A Pilot Study**. *Acute Crit Care* 2018, **33**(1):16-22.
39. Zulbaran-Rojas A, Mishra R, Rodriguez N, Bara RO, Lee M, Bagheri AB, Herlihy JP, Siddique M, Najafi B: **Safety and efficacy of electrical stimulation for lower-extremity muscle weakness in intensive care unit 2019 Novel Coronavirus patients: A phase I double-blinded randomized controlled trial**. *Front Med (Lausanne)* 2022, **9**:1017371.
40. Akar O, Gunay E, Sarinc Ulasli S, Murat Ulasli A, Kacar E, Sariaydin M, Solak O, Celik S, Unlu M: **Efficacy of neuromuscular electrical stimulation in patients with COPD followed in intensive care unit**. *The Clinical Respiratory Journal 2017 Nov;11(6):743-750* 2017.
41. Bao W, Yang J, Li M, Chen K, Ma Z, Bai Y, Xu Y: **Prevention of muscle atrophy in ICU patients without nerve injury by neuromuscular electrical stimulation: a randomized controlled study**. *BMC Musculoskelet Disord* 2022, **23**(1):780.
42. Dos Santos FV, Cipriano G, Jr., Vieira L, Güntzel Chiappa AM, Cipriano GBF, Vieira P, Zago JG, Castilhos M, da Silva ML, Chiappa GR: **Neuromuscular electrical stimulation combined with exercise decreases duration of mechanical ventilation in ICU patients: A randomized controlled trial**. *Physiother Theory Pract* 2020, **36**(5):580-588.
43. Guerra-Vega P, Guzmán R, Betancourt C, Grage M, Vera C, Artigas-Arias M, Muñoz-Cofré R, Vitzel KF, Marzuca-Nassr GN: **Medium-Frequency Neuromuscular Electrical Stimulation in Critically Ill Patients Promoted Larger Functional Capacity Improvement During Recovery than Low-Frequency Neuromuscular Electrical Stimulation: Randomized Clinical Trial**. *J Clin Med* 2025, **14**(15).
44. Othman SY, Elbiaa MA, Mansour ER, El-Menshawy AM, Elsayed SM: **Effect of neuromuscular electrical stimulation and early physical activity on ICU-acquired weakness in mechanically ventilated patients: A randomized controlled trial**. *Nurs Crit Care* 2024, **29**(3):584-596.
